# Who Is Willing to Engage in Social Gatherings During the Coronavirus Disease 2019 Lockdown? A Curvilinear Relationship Between Age and Heuristic Processing

**DOI:** 10.3389/fpsyg.2020.586408

**Published:** 2020-12-03

**Authors:** Kexin Wang, Siyue Li

**Affiliations:** College of Media and International Culture, Zhejiang University, Hangzhou, China

**Keywords:** age, heuristic processing, COVID-19, risky behavioral willingness, curvilinear relationship

## Abstract

**Background:**

The Chinese government implemented a lockdown to contain the coronavirus disease 2019 (COVID-19) pandemic during the Chinese Lunar New Year when people have the tradition to visit families and friends. Previous research suggested that heuristic processing increased risky behavioral willingness (e.g., desire to have social gatherings despite the pandemic) and that people’s tendency to use heuristic processing varied across different adulthood stages. This study thus investigated the relationships among age, heuristic processing of COVID-19-related information, and the willingness to have social gatherings during the lockdown.

**Methods:**

A sample of 1,651 participants was recruited from an online crowdsourcing platform between January 31 and February 04 in 2020, with a mean age of 30.69, 47.9% being women. Participants completed an online questionnaire about heuristic processing of COVID-19-related information, willingness to engage in social gatherings during the lockdown, age, and other demographic information.

**Results:**

Age was found to have a U-shaped curvilinear relationship with heuristic processing, and heuristic processing was positively correlated with the willingness to have social gatherings. Further analyses showed that heuristic processing curvilinearly mediated the relationship between age and the willingness to have social gatherings.

**Conclusion:**

Compared with young adults, emerging and older adults are more likely to engage in heuristic processing, which in turn, increases the willingness to have social gatherings. Heuristic processing serves as an underlying mechanism to explain the relationship between age and risky behavioral willingness.

## Introduction

The rapidly spreading coronavirus disease 2019 (COVID-19) has posed unprecedented threat to the public and has caused large-scale negative impacts around the world. In addition to medical efforts to contain the coronavirus, social and behavioral science can help mitigate the massive impacts by examining people’s psychological responses and health behaviors when faced with a global health crisis. For instance, Van Bavel et al. (2020) have noted that social and behavioral issues such as threat perceptions and coping in the context of COVID-19 deserve research attention.

Shortly after confirming that the virus can be transmitted through personal contact, the Chinese government launched a lockdown in Wuhan and advised the public to limit movements in January 2020. The outbreak, unfortunately, happened very close to the time of the Chinese Lunar New Year, which usually follows a week of gathering and reunion of family and friends. Staying home could be particularly challenging during the holiday season, especially for people who value traditional holidays. Despite the severity of the outbreak and warning against going out, people may still vary in the perceived risk of COVID-19 and willingness to comply with the stay-at-home policy. Specifically, different ways to process information may affect people’s willingness to take risky behaviors such as attending social gathering albeit the health threat.

In particular, heuristic processing has been found to facilitate risky behavioral willingness (Stock et al., 2015) and that the tendency to use heuristic processing fluctuates with age (Besedeš et al., 2012). Moreover, the risk of contracting COVID-19 has been found to be age-related. According to the latest data in *Nature Medicine*, not only the mortality rate but also the risk of symptomatic infection increased with age by 4% per year among adults aged 30–60 years old (Wu et al., 2020). Therefore, it is critical to understand whether and how people differing in age process COVID-19-related information and the extent to which they comply with the stay-at-home policy when facing such an unprecedented hazard.

Regarding the relationship between age, heuristic processing, and risky behavioral willingness in general, two competing hypotheses have emerged. Research from the aging perspective indicates that people are more likely to process information heuristically as they get older during adulthood (Kim et al., 2005), which in turn, increases risky behavioral willingness. On the other hand, research from the developmental perspective argues that people are less likely to process information heuristically as they grow older toward adulthood (Jacques and Marcovitch, 2010), which in turn, inhibits risky behavior-taking.

Due to fluctuation of heuristic processing across life span (Weller et al., 2011), the associations among age, heuristic processing, and risky behavioral willingness may not be fully represented by a linear relationship. However, little research has theorized non-linear relationships among the three variables and examined the heuristic processing as an explanatory mechanism. Therefore, the current study strives to investigate the curvilinear relationships among age, heuristic processing, and risky behavioral willingness, as well as to examine the mediating role of heuristic processing. By doing so, this research aims to provide an integrated and explanatory framework to understand how people of different ages process information and respond to a health crisis.

### Age and Heuristic Processing

Heuristic processing can be defined as using cognitive shortcuts to make decisions (Epstein, 1994) or “processing preconsciously, rapidly and effortlessly … feeling intuitively or self-evidently correct” (Klaczynski, 2000, p. 1348). Earlier research shows a positive association between age and heuristic processing (Johnson, 1990), arguing that older adults appear to use heuristic approaches more frequently than younger adults. More recent research, largely enlightened by the resource allocation theory, posits that social–cognitive functioning in adulthood is characterized by an age-related increase in selectivity (Hess, 2000). Specifically, cognitive capacity decreases with aging, leading to less effortful processing in older adults (Hess et al., 2001). In line with this reasoning, Kim et al. (2005) found that older adults spontaneously relied more on heuristic processing than younger adults when trying to make a decision. Using controlled experiments, Besedeš et al. (2012) reported that older adults indeed preferred heuristic processing than younger adults, resulting in worse decisions.

Unlike the aging perspective that claims a positive correlation between age and heuristic processing, developmental psychologists argue for the possibility of a negative correlation between age and heuristic processing among late teens and emerging adults. According to this perspective, heuristic processing, which is the “default” mode of the cognitive system (Klaczynski, 2001b), may decrease with age as the higher-order cognitive ability to control heuristic processing begins to develop from late teens (Jacobs and Klaczynski, 2002). Consistent with this reasoning, Klaczynski (2001a) reported that heuristic processing was negatively associated with age among adolescents and that middle adolescents relied less heavily on heuristics than early adolescents. Moreover, a recent study validated this cognitive evolution process, indicating that heuristic processing declines with age, with college students using less heuristic processing than adolescents (Barash et al., 2019).

Taken together, the aging perspective suggests a positive relationship between age and heuristic processing, whereas the developmental perspective posits a negative relationship between these two variables. Although the two perspectives are seemingly contradictory to one another, the positive and negative paths observed in previous research likely emerge at different stages of life and thus exhibit a U-shaped curve throughout time. In fact, Strough et al. (2011) found a negative association between age and heuristic processing among younger adults but a positive association among older adults. The results showed support for both the developing perspective and the aging perspective but at different stages of life. Likewise, Weller et al. (2011) noted that depending on the age stage, both positive and negative associations between age and heuristic processing might be valid. Recently, Barash et al. (2019) found that young adults prefer deliberate processing over heuristic processing, indicating their cognitive abilities and working memory are at the peak. However, the higher-order cognitive ability for teens has not fully developed to maturity, and the working memory begins to decline from middle adulthood.

Although prior research suggested a possibility of a U-shaped relationship between age and heuristics (e.g., Barash et al., 2019), to our best knowledge, no empirical study has explicitly investigated this curvilinear relationship. The first hypothesis was thus proposed to examine the complex relationship between age and heuristics.

H1 : The relationship between age and heuristic processing will have a U-shaped curve, with a negative association among emerging and young adults but a positive association among older adults.

### Heuristic Processing and Risky Behavioral Willingness

Heuristic processing is evidenced to increase judgmental errors and reasoning bias (Tversky and Kahneman, 1974; Lopes, 1991; Strough et al., 2011). Although heuristic processing can be efficient and even creative in some social domains such as cooperation and altruistic behaviors (Epstein, 1994; Capraro, 2019), this type of processing in itself is not very adaptive (Klaczynski, 2001b). Especially when estimating the probability of encountering a risky event (e.g., a health threat), using heuristic processing tends to cause underestimation of risk (Agans and Shaffer, 1994; Strough et al., 2011). For instance, when investigating a suspected cancer cluster’s judgment, heuristic processing of relevant information is associated with the judgment of lower risks (Trumbo, 1999, 2002). Klaczynski (2005) also pointed out that heuristic processing may lead to optimistic bias and make people underestimate the risk of cancer. At the same time, heuristic processing may also enhance the likelihood of risky behavior-taking through emotional arousals (Capraro and Barcelo, 2020b). Capraro and Barcelo (2020a, b) conducted a series of studies examining people’s intention to wear a face mask during the pandemic. Their results showed that people who experienced more negative emotions regarding health interventions were less willing to wear a face mask. Moreover, people who were primed to process information *via* emotions were less likely to wear a face mask compared with those primed to make decisions using reasoning.

Given that heuristic processing is associated with underestimation of potential risk, people prone to heuristic processing may perceive lower risk of attending social gatherings and report greater willingness to participate in such events. In addition, behavioral willingness “involves relatively little forethought, which means less consideration of outcomes or consequences” (Gibbons et al., 1998, p. 321). Therefore, (risky) behavioral willingness is highly likely to be facilitated by heuristic processing (Gerrard et al., 2008; Gibbons et al., 2010). In line with this reasoning, Stock et al. (2015) found that heuristics could increase the willingness to take unprotected sex. More importantly, heuristics “discard important information” (Besedeš et al., 2012) and lead people to arrive at a judgment based on existing knowledge or stereotypes (Trumbo, 1999). The existing knowledge, however, may not be applicable to this new context. In fact, even scientific knowledge about COVID-19 was far from enough at the onset of the outbreak. When people evaluate the risk of contracting COVID-19 based on previous knowledge about pneumonia or influenza, they are prone to underestimate the risk. Because heuristic processing drives people to underestimate risks, those engaging in more heuristic processing of COVID-19 information tend to show greater willingness to attend social gatherings during the pandemic. The second hypothesis was proposed:

H2 : Heuristic processing is positively associated with the willingness to attend social gatherings during the pandemic.

### Age–Heuristic Processing–Willingness to Engage in Social Gatherings

Previous research acknowledged the mediating role of heuristic processing in explaining the relationship between age and decision-making. For instance, Besedeš et al. (2012) suggested heuristics explained age differences in general decision-making. Henninger et al. (2010) demonstrated that cognitive processing (i.e., speed and memory) fully explained the age differences in risky decision-making.

Taking a step further, extant research suggests the likelihood of a curvilinear mediating role of heuristics in explaining the relationship between age and willingness to engage in social gatherings. From emerging (i.e., when heuristic processing decreases, Klaczynski, 2001a) adulthood to young adulthood, cognitive abilities tend to develop to its peak and inhibit reactive heuristic processing (Jacques and Marcovitch, 2010). A decrease in reliance on heuristic processing likely reduces people’s willingness to take risky behaviors (Gerrard et al., 2008). On the other hand, heuristic processing may increase with age from mid adulthood to late adulthood. During this age range, people may possess limited cognitive resources and rely more heavily on selective and stereotypical processing (Epstein, 1994), leading to more willingness to take risky behaviors (Stock et al., 2015). Thus, the associations among age, heuristic processing, and risky behavioral willingness may run as two parts, depending on the age range. Among emerging and young adults, heuristic processing is likely to decrease with age, which in turn, is related to less willingness to attend social gatherings. Among middle-aged and older adults, heuristic processing is likely to increase with age, which in turn, is related to more willingness to attend social gatherings. With the two parts taken together, we proposed that:

H3 : heuristic processing will mediate the association between age and willingness to attend social gatherings curvilinearly.

## Materials and Methods

### Sample and Procedures

From 31 January to February 04, 2020, a sample of 1,651 participants was recruited from an online crowdsourcing platform Sojump^1^. The platform has a total number of 2.6 million members from all regions of mainland China. Through sample service, members received an invitation email briefly introducing our research titled “COVID-19 and life experience.” Members who agreed to participate filled out the survey online and received a small amount of cash bonus after completion. Two attention check items were used in screening careless responses. Overall, data from 101 participants were deleted due to failure to pass the attention checks (*n* = 78, 4.72%) or duplicate cases from the same IP address (*n* = 23, 1.39%), leaving a total of 1,550 cases for final analysis.

The final sample had a mean age of 30.69 (*SD* = 8.31), with 49.7% (*n* = 770) being women. Most respondents were highly educated with 79.9% (*n* = 1238) having completed a Bachelor’s or higher degree. Besides, 3.7% (*n* = 58) of the respondents reported having worked or traveled in Wuhan in the past month.

### Measures

#### Heuristic Processing

Heuristic processing was measured by four items adapted from heuristic-related items in an information processing scale (Yang et al., 2014), such as “while reading the epidemic information, I focused on only a few points” and “I did not spend much time thinking about the epidemic information.” Respondents rated each item on a five-point Likert scale ranging from 1 = completely disagree to 5 = completely agree. An exploratory factor analysis (EFA) contained a one-factor construct that accounts for 56.75% of the total variance. The factor loading ranged from 0.72 to 0.80 (α = 0.74) for the four items. An average score was created with a higher value reflecting a higher level of heuristic processing (*M* = 2.18, *SD* = 0.72).

#### Willingness to Engage in Social Gatherings

Drawing upon the literature of risky behavior willingness, a scale from the study of Chen et al. (2019) was modified to measure the willingness to engage in social gatherings in the context of the COVID-19 pandemic during the Chinese Lunar New Year. Seven items were used to measure respondents’ willingness to engage in different types of social gatherings (e.g., “Your friends or relatives ask you to dine out during the COVID-19 outbreak, you think you can protect yourself, so you go.”). The items were assessed on a five-point Likert scale ranging from 1 = very unlikely to 5 = very likely. An EFA contained a one-factor construct that accounts for 52.23% of the total variance. The factor loading ranged from 0.65 to 0.77 (α = 0.85) for the seven items. An average score was created with a higher value reflecting a higher level of willingness to engage in social gatherings (*M* = 1.81, *SD* = 0.67).

#### Control Variables

Respondents answered questions about age, gender, educational level, travel/work history to Wuhan in the past month, current health condition, source, and consumption of COVID-19 information. Current health condition was measured by the Comorbidity Assessment Instrument (Bayliss et al., 2005), asking respondents to rate on a disease burden checklist from a five-point Likert scale ranging from 1 = not at all to 5 = a lot. A higher score reflected a poorer health condition. Source and consumption of COVID-19 information were measured by three questions, asking the frequency of consuming pandemic information from (1) central government media, (2) local government media, and (3) social media (1 = never, 5 = always).

### Analytical Strategy

We used the PROCESS macro for SPSS (model 4) with bootstrapping to analyze the data (Hayes, 2017). The model included all the control variables as covariates. The quadratic age term was entered as the independent variable to examine the hypothesized U-shaped curve. To reduce multicollinearity concern, age was standardized before creating its quadratic term. The linear age term was also controlled as a covariate. This PROCESS macro estimates the direct and indirect effects with a bootstrap procedure. When the confidential interval (95% CI, 5,000 samples) of indirect effect does not include zero, a mediating effect is significant. The two-lines analysis was conducted to provide more robust evidence for the U curve, as Simonsohn (2018) suggested that there should be both a significant decrease and a significant increase for a U curve.

## Results

### Preliminary Analysis

Table 1 presents the sample characteristics and Table 2 presents the means, standard deviations, and zero-order correlations among the key variables. Notably, the small and non-significant zero-order correlation between linear age term and heuristic processing (*r* = 0.01, *p* > 0.05) suggested that a curvilinear relationship should be considered.

**TABLE 1:** Sample characteristics (*N* = 1,550).

**Number of participants**	**1,550**
Female, *n* (%)	770 (49.7)
**Age group (years), *n* (%)**	
18–25	442 (28.5)
26–33	651 (42.0)
34–41	299 (19.3)
42–49	102 (6.6)
≥50	56 (3.6)
**Education, *n* (%)**	
Less than a high school diploma	17 (1.1)
High school degree	83 (5.3)
Associate degree	212 (13.7)
Bachelor’s degree	1,092(70.5)
Master’s degree	136 (8.8)
Doctorate degree	10 (0.6)
**Total household income per month, *n* (%)**	
≤1,000 RMB	48 (3.1)
1,001–3,000 RMB	74 (4.8)
3,001–5,000 RMB	150 (9.7)
5,001–8,000 RMB	256 (16.5)
8,001–10,000 RMB	227 (14.6)
10,001–15,000 RMB	335 (21.6)
15,001–20,000 RMB	245 (15.8)
20,001–50,000 RMB	194 (12.5)
≥50–000 RMB	21 (1.4)

**TABLE 2:** Descriptive statistics.

	***M***	**SD**	**1**	**2**	**3**	**4**	**5**	**6**	**7**	**8**	**9**
(1) Gender	−	−	1								
(2) Educational level	6.78	0.87	0.04	1							
(3) Travel/wok history to Wuhan	−	−	–0.03	−0.06*	1						
(4) Health condition	1.48	0.55	0.00	0.00	–0.01	1					
(5) CGM information consumption	4.17	0.99	–0.02	0.08**	–0.01	−0.05*	1				
(6) LGM information consumption	3.61	1.07	0.02	0.06**	0.03	−0.08**	0.47***	1			
(7) SM information consumption	4.45	0.78	0.07**	0.09**	–0.01	−0.11***	0.22***	0.20***	1		
(8) Age (linear term)	30.69	8.31	−0.16***	−0.13***	0.06*	0.11***	0.00	0.09***	−0.10***	1	
(9) Heuristic processing	2.18	0.72	–0.04	−0.10***	–0.05	0.07*	−0.20***	−0.18***	−0.17***	–0.01	1
(10) Willingness to engage in social gatherings	1.81	0.67	−0.09***	−0.10***	0.03	0.20***	−0.10***	−0.10***	−0.15***	−0.07*	0.17***

*N = 1,550. CGM, central government media; LGM, local government media; SM, social media. ^∗^p < 0.05, ^∗∗^p < 0.01, ^∗∗∗^p < 0.001.*

### Age and Heuristic Processing

**FIGURE 1:**
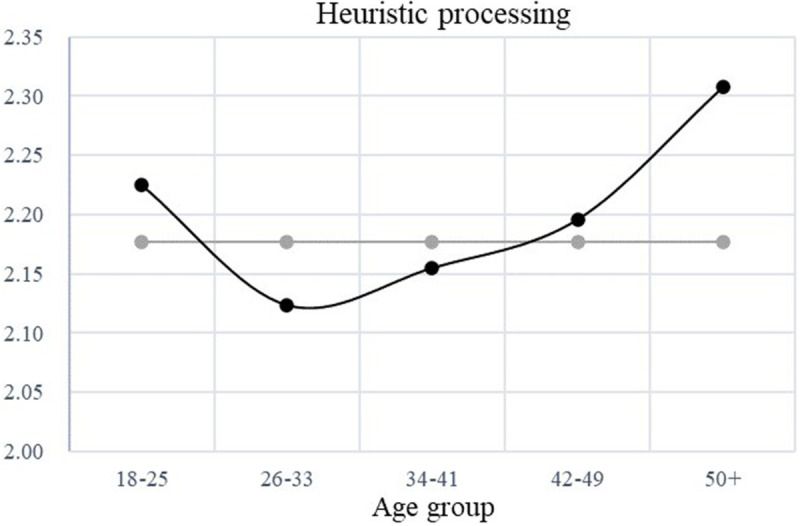
Curvilinear association between age and heuristic processing.

Table 3 presents the results of the hierarchical regression and mediation effect. H1 proposed that the association between age and heuristic processing would follow a U-shaped curve. The coefficient of the quadratic age term on heuristic processing reached significance (*B* = 0.046, *SE* = 0.012, *p* < 0.001, 95% CI [0.022, 0.071]), and the positive value indicated the presumed U curve appeared. In addition, the two-lines analysis validated this curve. As illustrated in Figure 1, the left part of the curve demonstrated a negative association between age and heuristic processing (*B*_*two–lines test*_ = −0.02, *z* = −3.18, *p* < 0.01), suggesting that from emerging adulthood to early adulthood (i.e., from 18 to roughly 30 years old), people were less likely to use heuristic processing as they grew older. The right part of the curve, however, demonstrated a positive association between age and heuristic processing (*B*_*two–lines test*_ = 0.01, *z* = 2.15, *p* < 0.05), showing that from middle adulthood to late adulthood (around 40–65 years old), people were more likely to use heuristic processing as people grew older. Notably, the magnitude of the positive slope increased for people over 50, indicating that people rely more heavily on heuristic processing toward their late adulthood. Thus, H1 was supported.

**TABLE 3:** Hierarchical regression and mediation effect.

	**Heuristic processing**				**Willingness to engaging in social gatherings**			
**Predictors**	***B***	***SE***	**LLCI**	**ULCI**	***B***	***SE***	**LLCI**	**ULCI**
Constant	4.117***	0.277	3.530	4.617	2.412***	0.275	1.872	2.952
Gender	–0.042	0.036	–0.112	0.028	−0.125***	0.033	–0.190	–0.060
Educational level	−0.052*	0.021	–0.093	–0.010	−0.063*	0.020	–0.101	–0.024
Travel/wok history to Wuhan	−0.195*	0.093	–0.377	–0.012	0.130	0.090	–0.039	0.298
Health condition	0.062	0.032	–0.002	0.125	0.232***	0.030	0.174	0.291
CGM information consumption	−0.093***	0.021	–0.133	–0.052	–0.025	0.019	–0.062	0.013
LGM information consumption	−0.052**	0.019	–0.089	–0.015	–0.007	0.018	–0.041	0.028
SM information consumption	−0.107***	0.023	–0.154	–0.061	−0.082***	0.022	–0.124	–0.039
Age (linear term)	−0.008**	0.003	–0.013	–0.003	−0.012***	002	–0.016	–0.007
Age (quadratic term)	0.046***	0.012	0.022	0.071	0.017	0.011	–0.006	0.039
Heuristic processing					0.110***	0.024	0.064	0.156
*R*^2^	0.082				0.104			
Δ*R*^2^	0.082***				0.022***			

Direct and indirect of age (quadratic term) on the willingness to engaging in social gatherings mediated by heuristic processing								

	**Effect**		**Boot *SE***		**Boot LLCI**		**Boot ULCI**	

Indirect effect	0.005		0.002		0.002		0.009	
Direct effect	0.017		0.011		−0.006		0.039	

*N = 1,550. CGM, central government media; LGM, local government media; SM, social media. ^∗^p < 0.05, ^∗∗^p < 0.01, ^∗∗∗^p < 0.001.*

### Heuristic Processing and Willingness to Engage in Social Gatherings

H2 proposed that heuristic processing was positively associated with the willingness to engage in social gatherings. As seen in Table 3, the heuristic processing coefficient on the willingness to engage in social gatherings reached significance and the positive value indicated that an increased heuristic processing was related to a higher level of behavioral willingness (*B* = 0.110, *SE* = 0.024, *p* < 0.001, 95% CI [0.064, 0.156]). Therefore, people who evaluated COVID-19-related information more spontaneously showed greater willingness to attend social gatherings. H2 was supported.

### Age, Heuristic Processing, and Willingness to Engage in Social Gatherings

**FIGURE 2:**
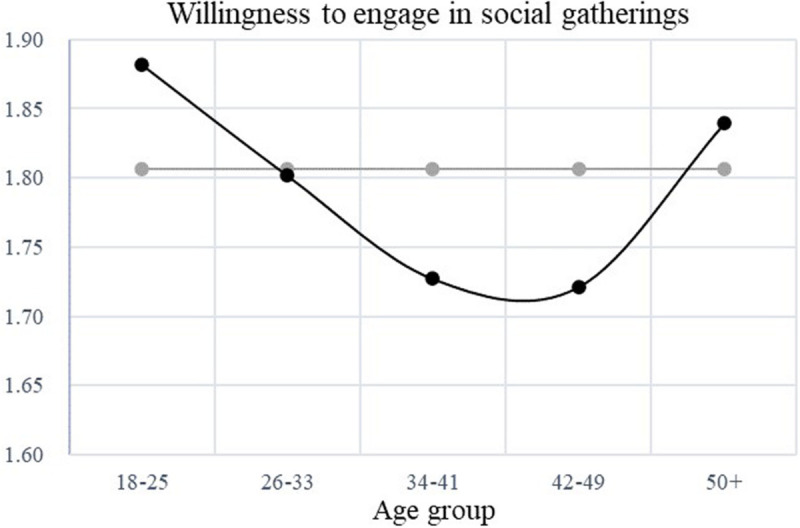
Curvilinear association between age and willingness to engaging in social gatherings.

H3 proposed that heuristic processing curvilinearly mediated the association between age and the willingness to engage in social gatherings. As shown in Table 3, the indirect effect reached significance and 95% CI did not include zero (indirect effect = 0.005, bootstrap *SE* = 0.002, 95% CI [0.002, 0.009]), suggesting that heuristic processing explained age differences in social gathering willingness. As such, H3 was supported. The findings were graphed in Figure 2 for ease of interpretation.

As shown in Figure 2, the left part of the curve depicted a negative relationship between age and the willingness to engage in social gatherings, showing that from emerging adulthood to middle adulthood (i.e., from 18 to roughly 40 years old), people were less willing to engage in social gatherings as they grew older. The negative relationship was explained by relatively less reliance on heuristic processing of COVID-19-related information. The right part of the curve, however, illustrated a positive relationship between age and the willingness to engage in social gatherings, suggesting that from middle adulthood to late adulthood (i.e., from around 45–65 years old), people were more inclined to engage in social gatherings as they grew older. This tendency could be explained by an increased reliance on heuristic processing when reading COVID-19-related information.

## Discussion

The COVID-19 pandemic has been causing a large-scale impact on individuals and the society across the globe. As the coronavirus is mainly transmitted through respiratory droplets from person to person, social distancing has been proved as an effective means to contain the pandemic (Lewnard and Lo, 2020). However, people differ in their willingness to keep social distance with others, especially during the Chinese Lunar New Year. This study examined whether the willingness to attend social gatherings during the pandemic was associated with age and also the possible mechanism underlying such a relationship. This study found support for both the aging perspective and developmental perspective, showing that age and willingness to attend social gatherings had a U-shaped relationship, with heuristic processing playing a mediating role in between the two variables. Specifically, this study found that (1) among emerging and young adults, people used less heuristic processing as they grew older, which in turn, decreased their willingness to engage in social gatherings during the lockdown; and (2) among middle-aged and older adults, people relied more heavily on heuristic processing as they grew older, which in turn, increased their willingness to engage in social gatherings.

### Hypothesized Model

Consistent with the developmental perspective which argues for a decline in heuristic processing from late teens (Klaczynski, 2005), this study found a negative association between age and heuristic processing among emerging and young adults. Compared with adults in their late 20s and early 30s, emerging adults are more likely to engage in a quick, effortless information processing and formulate their perceptions based on superficial and self-serving cues (Klaczynski, 2001a). The result suggests that a lack of careful evaluation of COVID-19 risk explains why emerging adults are still willing to dine out or meet friends during the lockdown. The decreased heuristic processing with age might be explained by metacognitive intercession which suggests that resistance to the default heuristic processing is not fully developed until early adulthood (Jacobs and Klaczynski, 2002). Research in neuroscience found the prefrontal lobes of the brain, which modulate basic attention and construct cognitive process, remain immature until the mid-20s (Casey et al., 2005).

Besides showing support for the developmental perspective, this study also lent support to the aging perspective by finding a positive association between age and heuristic processing among middle-aged and older adults. According to the resource allocation theory (Hess, 2000), older people are more selective in processing as they grow older because aging reduces cognitive capacity. In the context of a pandemic outbreak, older adults may perceive COVID-19 less threatening as prior research pointed that they tend to deliberatively ignore negative information when facing a potential threat (Gigerenzer and Garcia-Retamero, 2017). Therefore, older adults become more willing to involve in social gatherings than middle-aged adults. This finding could also be explained by the interference of long-held beliefs. Research shows that older people tend to hold many deeply ingrained beliefs which can facilitate heuristic processing (Klaczynski and Robinson, 2000). Because easily accessible information is more likely to be retrieved in heuristic processing (Kahlor et al., 2003), long-held beliefs which have been stored in memory for long come to mind quickly. People using heuristics may use long-held beliefs such as knowledge about a flu to make sense of COVID-19. As a result, older people may treat COVID-19 as a mild flu which may not pose severe threats to life, leading to increased risky behavior willingness.

### Implications

These findings have several theoretical and pragmatic implications. First, this study advances research on individual difference in decision-making by discovering a U-shaped curvilinear relationship between age and heuristic processing. Although prior research indicated the co-existence of negative and positive associations between age and heuristic processing (Strough et al., 2011; Barash et al., 2019), our study was the first to empirically test and validate the non-linear relationship. This finding reconciled the contradictory results in prior research which argued for either a positive or negative association between age and heuristic processing (Albert and Steinberg, 2011). Moreover, prior research on resource allocation theory suggested that a general shift in resource allocation occurs during adulthood as people may become more conservative in using cognitive resource from this stage (Hess et al., 2001). By analyzing age as a continuous spectrum, our finding indicated that the shift in resource allocation may occur around the 50s as the curve showed a noticeable rise around late 40s. In addition, we identified the explanatory role of heuristic processing in understanding age differences and risky behavioral willingness.

The findings showed that teens and adults over 50s were more likely to process information heuristically and thus be more willing to take risky behaviors than middle-aged people. Because heuristic processing may increase willingness to engage in risky behaviors, it is important to encourage people to thoughtfully process information instead of relying on heuristics. Motivating people to engage in deliberative processing might be an effective strategy. For instance, the elaboration likelihood model posits that enhancing personal relevance can prompt people to process information more systematically (Petty et al., 2003). In addition, the socioemotional selectivity theory stresses that positivity (compared to negativity) in information motivates older adults to pay more attention to the information as they seek to optimize the emotional experience in a later period of life (Peters et al., 2008; Strough et al., 2011). Guided by these theories, media practitioners and health professionals can emphasize the relevance of this pandemic to individuals and highlight the positive side of combating the pandemic to motivate more preventive behaviors such as social distancing. In addition, because older people are more susceptible to COVID-19 (Wu et al., 2020), more targeted risk communication is urgently needed to enhance people’s perceptions of risk and improve preventive intention, especially among the high-risk population such as the elderly people.

### Limitations and Future Research

Several limitations should be considered when interpreting these results. First, although we made efforts to cover a wide range of age groups, adolescents and elderly people were underrepresented. However, previous research indicated that early adolescents (mean age 12.4 years old) use more heuristic processing and take more risks than late adolescents (mean age 16.3 years old, Klaczynski, 2001a) and that adults who are 65–85 years old take more risks than adults who are 44–64 years old (Weller et al., 2011). It is thus reasonable to assume that the U curves found in our research are likely to present in a sample with a broader age range. Future research should try to collect data from a nationally representative sample to further test these U curves among age, heuristics, and risky behavioral willingness.

Second, the cross-sectional nature of this design refrains any causal inference. Although it is more logical to infer that cognition (e.g., heuristic processing) takes place prior to behavioral willingness (e.g., risky behavioral willingness) and a recent study has found that priming reasoning increases the intention to wear a face covering (Capraro and Barcelo, 2020b), we cannot rule out the possibility that the willingness to engage in social gatherings drives people to engage in more heuristic thinking. That is, as people are advised to stay at home during the pandemic, they may grow a strong desire to attend social gatherings. Future research may consider the reciprocal association between heuristic processing and risky behavioral willingness.

Third, the measurement for the willingness to engage in social gatherings is context specific and may not be applied to countries that do not celebrate the Chinese Lunar New Year. In addition, besides avoiding going out, many other preventive behaviors such as washing hands and wearing a mask have been found to be effective ways to cope with the pandemic (Wang et al., 2020). It is thus important for researchers to examine people’s willingness or resistance to take other preventive behaviors.

Fourth, this study did not control for infection rate in one’s neighborhood as this data was not available in the very early stage of the outbreak. Awareness of infection in one’s social surroundings, including family, friends, and local communities, was found to be associated with engagement in preventive behaviors during COVID-19 (Li et al., 2020). Therefore, future research may take into account infection rate when investigating people’s willingness to attend social gatherings during a pandemic.

Lastly, this study only considered heuristic processing as a cognitive factor that would explain age differences in the willingness to take risks during the pandemic. Recent research on COVID-19, however, has shown that other important cognitive and affective factors (e.g., anxiety, optimism, and fear of death) would impact risk perception and explain age differences in subsequent protective behaviors (Pasion et al., 2020).

## Conclusion

This study examined if and how people with different ages vary in their willingness to attend social gatherings during the outbreak of COVID-19 pandemic in China. The results suggest a U-shaped pattern between age and willingness to attend social gatherings, mediated by heuristic processing of COVID-19 information. This pattern explained why emerging adults and older adults, compared with middle-aged adults, are more willing to take risks to engage in social gatherings despite the health threat. The age–cognitive processing–behavioral willingness pattern provides insights for future research on public health communication and prevention.

## Data Availability Statement

The raw data supporting the conclusions of this article will be made available by the authors, without undue reservation.

## Ethics Statement

The studies involving human participants were reviewed and approved by Zhejiang University. The patients/participants provided their written informed consent to participate in this study.

## Author Contributions

KW designed the study, conducted the analysis, and drafted the manuscript. SL designed the study, interpreted the data, and helped to draft the manuscript. Both authors read and approved the final version of the manuscript.

## Conflict of Interest

The authors declare that the research was conducted in the absence of any commercial or financial relationships that could be construed as a potential conflict of interest.

## Footnotes

1 https://www.wjx.cn

## References

[B1] Agans R. P. Shaffer L. S. (1994). The hindsight bias: the role of the availability heuristic and perceived risk. *Basic Appl. Soc. Psychol.* 15 439–449. 10.1207/s15324834basp1504_3

[B2] Albert D. Steinberg L. (2011). Judgment and decision making in adolescence. *J. Res. Adolesc.* 21 211–224. 10.1111/j.1532-7795.2010.00724.x

[B3] Barash J. Brocas I. Carrillo J. D. Kodaverdian N. (2019). Heuristic to Bayesian: the evolution of reasoning from childhood to adulthood. *J. Econ. Behav. Organ.* 159 305–322. 10.1016/j.jebo.2018.05.008

[B4] Bayliss E. A. Ellis J. L. Steiner J. F. (2005). Subjective assessments of comorbidity correlate with quality of life health outcomes: initial validation of a comorbidity assessment instrument. *Health Q. Life Outcom.* 3 51–58. 10.1186/1477-7525-3-51 16137329 PMC1208932

[B5] Besedeš T. Deck C. Sarangi S. Shor M. (2012). Age effects and heuristics in decision making. *Rev. Econ. Statist.* 94 580–595. 10.1162/REST_a_00174 PMC3337688 22544977

[B6] Capraro V. (2019). The dual-process approach to human sociality: a review. *PsyarXiv* [Preprint], 10.31234/osf.io/432yw

[B7] Capraro V. Barcelo H. (2020a). Priming reasoning increases intentions to wear a face covering to slow down COVID-19 transmission. *arXiv* [Preprint], 10.31234/osf.io/wtcqy PMC8013666 33821089

[B8] Capraro V. Barcelo H. (2020b). The effect of messaging and gender on intentions to wear a face covering to slow down COVID-19 transmission. *PsyarXiv [Preprint]*. Available online at: https://psyarxiv.com/tg7vz 10.1002/acp.3793 PMC8013666 33821089

[B9] Casey B. J. Tottenham N. Liston C. Durston S. (2005). Imaging the developing brain: what have we learned about cognitive development? *Trends Cogn. Sci.* 9 104–110. 10.1016/j.tics.2005.01.011 15737818

[B10] Chen S. Schreurs L. Pabian S. Vandenbosch L. (2019). Daredevils on social media: a comprehensive approach toward risky selfie behavior among adolescents. *New Media Soc*. 21, 2443–2462. 10.1177/1461444819850112

[B11] Epstein S. (1994). Integration of the cognitive and the psychodynamic unconscious. *Am. Psychol.* 49 709–724. 10.1037/0003-066X.49.8.709 8092614

[B12] Gerrard M. Gibbons F. X. Houlihan A. E. Stock M. L. Pomery E. A. (2008). A dual-process approach to health risk decision making: the prototype willingness model. *Dev. Rev.* 28 29–61. 10.1016/j.dr.2007.10.001

[B13] Gibbons F. X. Etcheverry P. E. Stock M. L. Gerrard M. Weng C.-Y. Kiviniemi M. (2010). Exploring the link between racial discrimination and substance use: what mediates? What buffers? *J. Pers. Soc. Psychol.* 99 785–801. 10.1037/a0019880 20677890 PMC3314492

[B14] Gibbons F. X. Gerrard M. Ouellette J. A. Burzette R. (1998). Cognitive antecedents to adolescent health risk: discriminating between behavioral intention and behavioral willingness. *Psychol. Health* 13, 319–339. 10.1080/08870449808406754

[B15] Gigerenzer G. Garcia-Retamero R. (2017). Cassandra’s regret: the psychology of not wanting to know. *Psychol. Rev.* 124 179–196. 10.1037/rev0000055 28221086

[B16] Hayes A. F. (2017). *Introduction to Mediation, Moderation, and Conditional Process Analysis: A Regression-Based Approach.* New York, NY: Guilford Publications.

[B17] Henninger D. E. Madden D. J. Huettel S. A. (2010). Processing speed and memory mediate age-related differences in decision making. *Psychol. Aging* 25 262–270. 10.1037/a0019096 20545412 PMC2896211

[B18] Hess T. M. (2000). “Aging-related constraints and adaptations in social information processing,” in *Generative Mental Processes and Cognitive Resources*, eds von Hecker U. Dutke S. Sedek G. (Dordrecht: Springer), 129–155.

[B19] Hess T. M. Rosenberg D. C. Waters S. J. (2001). Motivation and representational processes in adulthood: the effects of social accountability and information relevance. *Psychol. Aging* 16 629–642. 10.1037/0882-7974.16.4.629 11766917

[B20] Jacobs J. E. Klaczynski P. A. (2002). The development of judgment and decision making during childhood and adolescence. *Curr. Direct. Psychol. Sci.* 11 145–149. 10.1111/1467-8721.00188

[B21] Jacques S. Marcovitch S. (2010). *Development of Executive Function Across the Life Span.* Hoboken, NJ: Wiley.

[B22] Johnson M. M. (1990). Age differences in decision making: a process methodology for examining strategic information processing. *J. Gerontol.* 45 75–78. 10.1093/geronj/45.2.P75 2179392

[B23] Kahlor L. Dunwoody S. Griffin R. J. Neuwirth K. Giese J. (2003). Studying heuristic-systematic processing of risk communication. *Risk Analys. Intern. J.* 23 355–368. 10.1111/1539-6924.00314 12731819

[B24] Kim S. Goldstein D. Hasher L. Zacks R. T. (2005). Framing effects in younger and older adults. *J. Gerontol. Ser. B Psychol. Sci. Soc. Sci.* 60 215–218. 10.1093/geronb/60.4.P215 15980289 PMC1751470

[B25] Klaczynski P. A. (2000). Motivated scientific reasoning biases, epistemological beliefs, and theory polarization: a two-process approach to adolescent cognition. *Child Dev*. 71, 1347–1366. 10.1111/1467-8624.00232 11108100

[B26] Klaczynski P. A. (2001a). Analytic and heuristic processing influences on adolescent reasoning and decision-making. *Child Dev.* 72 844–861. 10.1111/1467-8624.00319 11405586

[B27] Klaczynski P. A. (2001b). Framing effects on adolescent task representations, analytic and heuristic processing, and decision making: implications for the normative/descriptive gap. *J. Appl. Dev. Psychol.* 22 289–309. 10.1016/S0193-3973(01)00085-5

[B28] Klaczynski P. A. (2005). “Metacognition and cognitive variability: a dual-process model of decision making and its development,” in *The Development of Judgment and Decision Making in Children and Adolescents*, eds Jacobs J. E. Klaczynski P. A. (Mahwah, NJ: Erlbaum), 39–76.

[B29] Klaczynski P. A. Robinson B. (2000). Personal theories, intellectual ability, and epistemological beliefs: adult age differences in everyday reasoning biases. *Psychol. Aging* 15 400–416. 10.1037/0882-7974.15.3.400 11014705

[B30] Lewnard J. A. Lo N. C. (2020). Scientific and ethical basis for social-distancing interventions against COVID-19. *Lancet Infect. Dis.* 20 631–633. 10.1016/S1473-3099(20)30190-0 32213329 PMC7118670

[B31] Li S. Feng B. Liao W. Pan W. (2020). Internet use, risk awareness, and demographic characteristics associated with engagement in preventive behaviors and testing: cross-sectional survey on COVID-19 in the United States. *J. Med. Internet Res.* 22:e19782. 10.2196/19782 32501801 PMC7299540

[B32] Lopes L. L. (1991). The rhetoric of irrationality. *Theory Psychol.* 1 65–82. 10.1177/0959354391011005

[B33] Pasion R. Paiva T. O. Fernandes C. Barbosa F. (2020). The AGE effect on protective behaviors during the COVID-19 outbreak: sociodemographic, perceptions and psychological accounts. *Front. Psychol.* 11:561785. 10.3389/fpsyg.2020.561785 33178069 PMC7595956

[B34] Peters E. Diefenbach M. A. Hess T. M. Västfjäll D. (2008). Age differences in dual information-processing modes: implications for cancer decision making. *Cancer Interdiscipl. Intern. J. Am. Cancer Soc.* 113 3556–3567. 10.1002/cncr.23944 19058148 PMC2755493

[B35] Petty R. E. Wheeler S. C. Tormala Z. L. (2003). “Persuasion and attitude change,” in *Comprehensive Handbook of Psychology*, (2nd edn) eds Millon T. Lerner M. J. (New York, NY: John Wiley & Sons), 353–382.

[B36] Simonsohn U. (2018). Two lines: a valid alternative to the invalid testing of U-shaped relationships with quadratic regressions. *Adv. Methods Pract. Psychol. Sci.* 1 538–555. 10.1177/2515245918805755

[B37] Stock M. L. Gibbons F. X. Beekman J. B. Gerrard M. (2015). It only takes once: the absent-exempt heuristic and reactions to comparison-based sexual risk information. *J. Pers. Soc. Psychol.* 109:35. 10.1037/a0039277 26098587 PMC4477276

[B38] Strough J. Karns T. E. Schlosnagle L. (2011). Decision-making heuristics and biases across the life span. *Ann. N. Y. Acad. Sci.* 1235 57–74. 10.1111/j.1749-6632.2011.06208.x 22023568 PMC3755606

[B39] Trumbo C. W. (1999). Heuristic-systematic information processing and risk judgment. *Risk Analys.* 19 391–400. 10.1111/j.1539-6924.1999.tb00415.x 10765412

[B40] Trumbo C. W. (2002). Information processing and risk perception: an adaptation of the heuristic-systematic model. *J. Commun.* 52 367–382. 10.1111/j.1460-2466.2002.tb02550.x

[B41] Tversky A. Kahneman D. (1974). Judgment under uncertainty: heuristics and biases. *Science* 185 1124–1131. 10.1126/science.185.4157.1124 17835457

[B42] Van Bavel J. J. Baicker K. Boggio P. S. Capraro V. Cichocka A. Cikara M. (2020). Using social and behavioural science to support COVID-19 pandemic response. *Nat. Hum. Behav.* 4 460–471. 10.1038/s41562-020-0884-z 32355299

[B43] Wang C. Pan R. Wan X. Tan Y. Xu L. Ho C. S. (2020). Immediate psychological responses and associated factors during the initial stage of the 2019 coronavirus disease (COVID-19) epidemic among the general population in China. *Intern. J. Environ. Res. Public Health* 17:1729 10.3390/ijerph17051729 PMC7084952 32155789

[B44] Weller J. A. Levin I. P. Denburg N. L. (2011). Trajectory of risky decision making for potential gains and losses from ages 5 to 85. *J. Behav. Decis. Mak.* 24 331–344. 10.1002/bdm.690

[B45] Wu J. T. Leung K. Bushman M. Kishore N. Niehus R. de Salazar P. M. (2020). Estimating clinical severity of COVID-19 from the transmission dynamics in Wuhan, China. *Nat. Med.* 26 506–510. 10.1038/s41591-020-0822-7 32284616 PMC7094929

[B46] Yang Z. J. Rickard L. N. Harrison T. M. Seo M. (2014). Applying the risk information seeking and processing model to examine support for climate change mitigation policy. *Sci. Commu*. 36, 296–324. 10.1177/1075547014525350

